# Maximizing solar power generation through conventional and digital MPPT techniques: a comparative analysis

**DOI:** 10.1038/s41598-024-59776-z

**Published:** 2024-04-18

**Authors:** Shahjahan Alias Sarang, Muhammad Amir Raza, Madeeha Panhwar, Malhar Khan, Ghulam Abbas, Ezzeddine Touti, Abdullah Altamimi, Andika Aji Wijaya

**Affiliations:** 1 Department of Electrical Engineering, Mehran University of Engineering and Technology, SZAB Campus Khairpur Mir’s, Sindh, 66020 Pakistan; 2 School of Electrical Engineering, Southeast University, Nanjing, China; 3 Department of Electrical Engineering, College of Engineering, Northern Border University, 91431 Arar, Saudi Arabia; 4 Department of Electrical Engineering, College of Engineering, Majmaah University, 11952 Al-Majma’ah, Saudi Arabia; 5 Engineering and Applied Science Research Center, Majmaah University, 11952 Al-Majma’ah, Riyadh Saudi Arabia; 6 Mechanical Engineering Department, College of Engineering, University of Business and Technology, Jeddah, Saudi Arabia

**Keywords:** Conventional MPPTs, Artificial intelligence MPPTs, Solar energy, Sustainability, Energy science and technology, Engineering, Electrical and electronic engineering, Energy infrastructure

## Abstract

A substantial level of significance has been placed on renewable energy systems, especially photovoltaic (PV) systems, given the urgent global apprehensions regarding climate change and the need to cut carbon emissions. One of the main concerns in the field of PV is the ability to track power effectively over a range of factors. In the context of solar power extraction, this research paper performs a thorough comparative examination of ten controllers, including both conventional maximum power point tracking (MPPT) controllers and artificial intelligence (AI) controllers. Various factors, such as voltage, current, power, weather dependence, cost, complexity, response time, periodic tuning, stability, partial shading, and accuracy, are all intended to be evaluated by the study. It is aimed to provide insight into how well each controller performs in various circumstances by carefully examining these broad parameters. The main goal is to identify and recommend the best controller based on their performance. It is notified that, conventional techniques like INC, P&O, INC-PSO, P&O-PSO, achieved accuracies of 94.3, 97.6, 98.4, 99.6 respectively while AI based techniques Fuzzy-PSO, ANN, ANFIS, ANN-PSO, PSO, and FLC achieved accuracies of 98.6, 98, 98.6, 98.8, 98.2, 98 respectively. The results of this study add significantly to our knowledge of the applicability and effectiveness of both AI and traditional MPPT controllers, which will help the solar industry make well-informed choices when implementing solar energy systems.

## Introduction

All developed and underdeveloped nations can utilize renewable natural resources such as sunlight, wind, water, and geothermal heat by utilizing renewable energy technologies. They can generate electricity, heat buildings and commercial spaces, and power automobiles. Since renewable energy technologies don't emit greenhouse gases or other pollutants, they are better for the environment than conventional fossil fuels^1^. They also aid in lowering our dependency on imported energy. Some of the most popular renewable energy sources are solar, wind, hydro, geothermal, and biomass energy. PV solar power systems have the potential to contribute significantly to supplying the world's energy demands in the future. They create zero emissions of greenhouse gases and are clean, renewable energy sources. This makes it a wise decision to lessen our reliance on fossil fuels and slow down global warming. Systems using solar photovoltaic energy are also getting cheaper and more effective. The cost of solar panels has dropped significantly in recent years, and the efficiency of solar cells has also grown^2^. Now, solar photovoltaic systems can generate more power for a lower cost. PV solar energy systems are not only reasonably priced and effective but also incredibly adaptable. Installation options include the roof, the ground, and even the water. This implies that they may be applied in many settings, regardless of temperature or topography. Solar photovoltaic systems have a wide range of benefits. They can aid in lowering greenhouse gas emissions, dependency on fossil fuels, and energy costs^3^. During power outages, they can also offer backup power. The potential for solar photovoltaic systems to significantly contribute to the global energy mix is expanding as solar photovoltaic technology advances and costs drop. Future residential, commercial, and transportation energy needs may be mostly met by solar power systems.

A solar PV system uses solar panels or cells to capture sunlight and turn it into electrical power. Solar panels and solar cells, which respond to photons, or solar energy particles, with various solar spectrum wavelengths, are made from semiconductor materials. A solar inverter, solar tracking system, battery, mounting, cabling, and electrical accessories are examples of additional components that solar PV systems could be included to enhance functionality and use. Direct Current (DC) power is produced in a photovoltaic system using solar panels, which absorb sunlight^4^. The inverter then converts the DC power into Alternating Current (AC) electricity that may be used in your residence or place of business. In addition, batteries are needed to store electrical energy in the event that the system is connected to the grid or directly uses generated electricity. Batteries are only required if the user demands electricity at night. Due to its sustainability once installed, PV systems have the benefit of low running costs. Because they are incredibly durable and designed to endure for many years, they require very little maintenance^5^. Lastly, they don't require any lubricants because they are mechanically motionless immobile systems. Despite being the most widely utilized kind of renewable energy, photovoltaic systems have a number of shortcomings that are being investigated and fixed^6^. Solar PV systems are dependent on sunlight to generate electricity. Therefore, Extreme weather events, rain, snow, and cloud cover all have an impact on how much power solar panels can produce. Although photovoltaic systems are the most widely used kind of renewable energy, they have a number of shortcomings that are being investigated and fixed. The fact that photovoltaic technology is so dependent on the weather, or more precisely, one of the most significant shortcomings of the technology, is the amount and direction of sunlight impacting the panel surface. It is, hence, erratic and unreliable^7^. Additionally, the photovoltaic system's conversion rate or efficiency is low when compared to other power-generating systems. A significant number of solar panels must be erected because a single solar panel's efficiency is low, and adding more solar panels would increase the required land area. For every system, especially complicated systems like solar PV systems where the variable solar irradiation causes voltage instability and frequency deviation, regulation and control are two fundamental building elements. There must be certain regulating techniques and monitoring systems in place to provide a dependable and effective supply from the solar PV system, which further drives up the cost^8^.

A solar PV array's performance and output can be significantly impacted by shading. The smooth passage of sunlight onto the surface of PV cells is disrupted when shadows fall on a solar panel. These shadows could be cast by nearby objects such as trees, buildings, or even debris. The effect of such shading is twofold: it reduces the overall irradiance reaching the shaded cells and introduces electrical bypass pathways^9^. The reduction in irradiance limits the amount of light available for conversion into electrical energy, ultimately lowering the power output of the shaded panels. Sunlight, normally uniform across the surface of the solar array, becomes fragmented, creating an uneven distribution of energy absorption. The impacted cells' ability to generate electricity is severely reduced as a result, which lowers the system's overall efficiency. Partial shading affects the MPPT algorithm's performance. The solar panel cannot get continuous sunshine because of weather fluctuations, climatic variations, and variations in the angle at which solar radiation strikes the panel. Therefore, it is essential to use an MPPT technique that can maximize solar panel power depending on the weather at the time. The shading issue affects the power and current versus voltage curves^10^.

**Figure 1:**
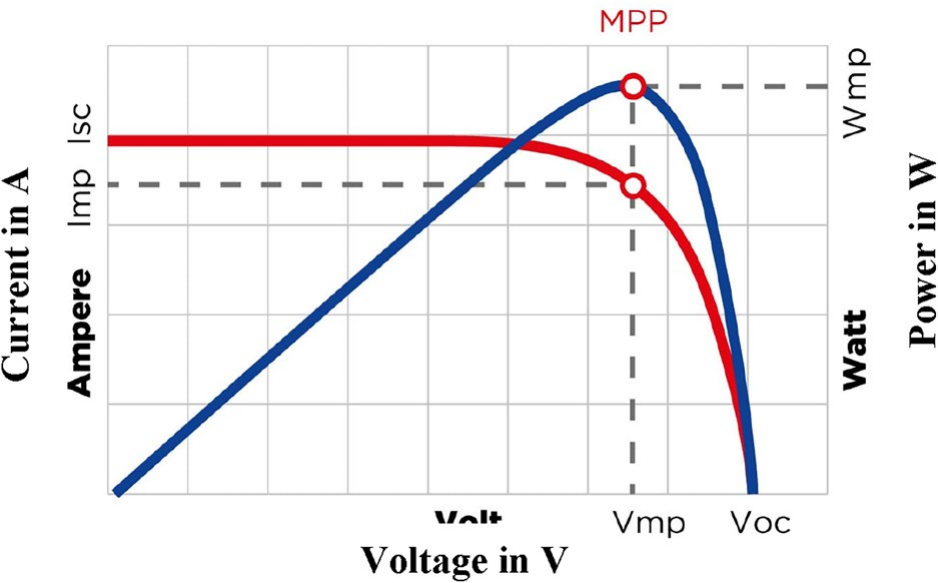
Graph showing power and voltage relationship^14^.

The MPPT method is used in PV systems to boost a solar panel's power output. It serves the purpose of ensuring that the solar panel is producing the highest amount of electrical power when it is functioning at its maximum power point (MPP), which is located on the current–voltage (I–V) curve^11^. The power output of solar panels fluctuates based on the operating conditions because of their non-linear I–V curve, as shown in Fig. 1. MPPT is employed in PV systems to boost overall efficiency and energy production. Temperature, shade, and the quantity of sunshine received are a few instances of variables influencing the MPP. MPPT algorithms continuously monitor the MPP by adjusting the operating voltage and current of the solar panel to extract the maximum amount of electricity possible^12^. PV systems employ MPPT to boost overall efficiency and energy output. Higher energy output may be achieved by running the solar panel at its MPP, which allows for greater power harvesting from the sun. This is especially important when the solar panel is connected to a battery or grid since it makes the best use of the solar energy that is currently available and improves the system's performance^13^.

This work aims to make a substantial contribution to the field of solar energy systems and control algorithms.

1. Specifically, it evaluates a highly advanced PV model for MPPT tacking.
2. Our focus extends to the rigorous evaluation of ten distinct MPPT controllers, including conventional methods such as INC, P&O, INC-PSO, P&O-PSO and advanced approaches employing AI such as ANN, FLC, ANFIS, PSO, ANN-PSO and FLC-PSO.
3. The crux of our contribution lies in the comprehensive comparative analysis of these controllers, assessing key performance parameters such as maximum output voltage, extracted maximum power, time response dynamics, design complexity, and system stability.
4. The ultimate goal of this research is to guide the scientific community in selecting and optimizing MPPT algorithms for improved solar energy harvesting.

## Related work on MPPT techniques

A state-of-the-art literature review is conducted to analyze the research gap and present the novelty of the proposed technique. The Study presents a novel MPPT method utilizing Artificial Neural Networks (ANN) to efficiently track the maximum power generated by a PV panel. The proposed ANN-based MPPT algorithm demonstrates rapid and accurate adaptation to changing meteorological conditions, including variations in temperature and solar radiation. Comprehensive research includes the design and modeling of a PV system structure in conjunction with the ANN-MPPT controller. The main goal of the study is to develop a high-performance ANN-based MPPT controller for solar applications.

In^15^, the authors introduced the Fuzzy Logic (FL) MPPT algorithm, a novel fuzzy logic-based method for monitoring the maximum power point of PV arrays. Unlike standard FL-MPPT methods that employ the change in slope of P–V characteristics, the proposed technique uses a new parameter termed "Ea" that is generated from I–V characteristics. This additional parameter improves tracking performance in a variety of environmental conditions (ECs) and increases the precision with which duty ratio changes may be computed. Using the "Ea" parameter, the approach successfully distinguishes between the operating point's placement in the Voltage Source Region (VSR) or Current Source Region (CSR) and its proximity to the MPP region.

Another study highlights the importance of MPPT controllers for optimizing the performance of solar (PV) modules^16^. The authors present a comparison of Adaptive Neuro-Fuzzy Inference System (ANFIS)-based MPPT controller architecture, an FL power controller, a PV module, an ANFIS reference model, and a DC-DC boost converter. Through simulations in the MATLAB/Simulink environment, the proposed ANFIS-based MPPT controller successfully harvests the maximum power from the PV module under a variety of weather circumstances, contributing significantly to the advancement of MPPT methods for solar energy systems.

Based on the literature study, the hardware implementation of the Incremental Conductance (INC) approach for MPPT in PV systems using Arduino boards is an important field of study in renewable energy. Numerous studies have examined the efficacy of MPPT techniques, and INC has emerged as a viable tool due to its ability to follow the MPP in quickly changing environmental settings. Researchers have investigated the integration of INC with DC-DC Boost converters, PV panels, and resistive loads in order to optimize energy harvesting in PV systems. Previous studies have also highlighted the importance of doing simulation trials using tools like MATLAB/Simulink to confirm the algorithm's performance prior to putting it on Arduino hardware. A strong experimental validation of INC-based MPPT controllers has been made possible by the incorporation of spatial Simulink packages, such as the "support package for Arduino hardware," despite the popularity of Arduino-based MPPT systems' low cost and accessibility. Overall, the research studies have concluded that the successful application of the INC algorithm in obtaining effective MPP extraction from PV panels will pave the way for its future use in renewable energy systems^17^.

The invention and improvement of MPPT algorithms, which are essential for effectively capturing the Global Maximum Power Point (GMPP) even in scenarios involving partial shade of PV arrays, is a key factor in improving the efficiency of PV systems. The Particle Swarm Optimization (PSO) algorithm stands out as a soft computing strategy among several MPPT methods. Hardware simplicity and independence from the installed PV system are two benefits of conventional PSO-based MPPT trackers. Nevertheless, choosing the parameters for the PSO to enable the successful extraction of the GMPP is a significant difficulty in real PSO deployment. The lack of a defined methodology for choosing the best parameters in PSO-based MPPT controllers for PV systems is revealed by an analysis of the current literature. The study conducted by^18^ closes this gap by putting forth a methodical and logical approach to determine the optimal PSO algorithm settings, accounting for factors such as solar panel arrangement, DC-DC converter topology, and even associated battery parameters. Furthermore, the work presents a novel method for determining the optimal sample period to maximize the performance of digital MPPT controllers. The modified PSO algorithm, along with its customized parameters, best satisfies the requirements of MPPT control for PV systems, representing a significant step towards increasing the overall effectiveness of such systems.

In the realm of PV arrays, tracking the MPP is one area where achieving greater energy conversion efficiency is always crucial. This objective is particularly crucial when partially shaded conditions exist. In these situations, the power–voltage (P–V) characteristic curve of PV arrays exhibits multiple peaks, making it challenging for conventional MPPT techniques to distinguish between the local MPPs and the global MPPs. Research on developing strategies for efficiently monitoring the GMPP while reducing the adverse effects of partial shadow has increased significantly. PSO, one of these methods, has become popular because of its quick tracking abilities and flexibility in various environmental circumstances. However, a number of changes and improvements have evolved to overcome several flaws in traditional PSO techniques. In^19^, the authors perform a comprehensive and comparative study of various PSO-based methods, considering significant factors such as particle initialization criteria, search space exploration, convergence speed, initial parameter settings, performance with and without partial shading, and overall efficacy. Comprehensive simulation tests utilizing MATLAB code and the Simulink package extensively evaluate the suitability of these methodologies for real PV systems running under varied operating circumstances.

In the work done by^20^, they investigate the behavior of a photovoltaic system under various environmental conditions, such as intermittent variations in the atmosphere. The primary objective is to improve the system's performance by contrasting two MPPT algorithms: the PSO and the P&O algorithm. The efficiency, stability, speed, and robustness of the algorithms are tested in a range of atmospheric conditions. Simulation data shows that the PSO algorithm outperforms the P&O approach, highlighting its superior efficiency in maximizing power generation under a variety of environmental situations. Their study provides insightful recommendations for enhancing the efficiency of solar systems and emphasizes the critical need for selecting the appropriate MPPT algorithms for better energy harvesting.

Another study by^21^ aims to enhance the performance of microgrid systems by creating a self-adapting energy management model that integrates optimal ANN. The proposed model is composed of a series of artificial neural networks that have been optimized individually through the application of PSO. The model aims to estimate and provide essential data to the energy management system to enhance the microgrid's integration of various energy sources. After being constructed in MATLAB/Simulink, the model is validated using experimental data.

A novel hybrid Fuzzy Particle Swarm Optimization (FPSO) technique, in conjunction with a photovoltaic-fed shunt active power filter, is proposed by^22^ to increase power quality and produce clean electricity. MPPT is a function of the FPSO system, which tracks the MPP and extracts as much energy as possible from the PV system. Fuzzy logic and synchronous reference frame theory govern the photovoltaic-fed shunt active power filter, which connects the boost converter output to the grid. The results demonstrate that the recommended controller performs well in a variety of load circumstances, resulting in improved power quality and more environmentally friendly electricity distribution.

Techniques for MPPT are essential for optimizing PV systems' efficiency and performance. The rapid expansion of the PV sector has led to the suggestion of various MPPT approaches. One of these, the INC approach, locates the MPP with a high rate of convergence, although it suffers from significant ripple under continuous radiation. In contrast, the PSO method has a slower rate of convergence than the INC method but a lower ripple output power. A new approach that integrates and combines the INC and PSO methodologies is proposed by^23^ in order to make use of the benefits of both approaches. This innovative method takes advantage of the INC method's rapid convergence in response to radiative changes and the PSO method's stability and excellent accuracy under conditions of continuous irradiation. The suggested strategy attempts to boost the MPPT performance for PV systems, increasing their general efficiency and stability, by utilizing these technologies in concert.

The numerous MPPT strategies used in solar systems are thoroughly examined in this literature review, which classifies them into conventional, intelligent, optimization, and hybrid methodologies. While offering simplicity and cost-effectiveness, traditional MPPT techniques like P&O and INC may have slow tracking and oscillations. Fuzzy logic and neural networks are intelligent ways that improve tracking speed and accuracy but may need a lot of processing power. High tracking efficiency is provided by optimization-based approaches like P&O with the Newton–Raphson method, although they can be computationally demanding. Hybrid methods, which combine components of many methodologies, offer a compromise between complexity and efficiency. Although hybrid methods are most effective, they are typically more complicated and expensive, making conventional and intelligent techniques appealing alternatives.

The availability of different methods presents issues for maintaining continuous power generation from solar PV systems and ensuring the usage of optimum MPPT controllers. As a result, a thorough comparison study is required. In line with current research trends in renewable energy, our study is to perform a thorough comparative analysis of these controllers by applying real environments and a variety of weather conditions^24^.

## Conventional MPPT techniques

Conventional MPPT methods are fundamental approaches used in solar energy system optimization with the goal of improving PV system efficiency. Of these, the most often used are INC and P&O due to their ease of use and integration. These methods work by dynamically modifying the PV system's operating point in order to track the MPP in a range of environmental circumstances. While INC is recognized for its rapid convergence and adaptability to dynamic changes, P&O offers a straightforward approach, albeit with potential accuracy challenges in certain scenarios^25^. Practitioners must have a thorough understanding of these traditional MPPT approaches in order to design and implement PV systems with the best possible energy extraction.

### Introduction to incremental conductance

**Figure 2:**
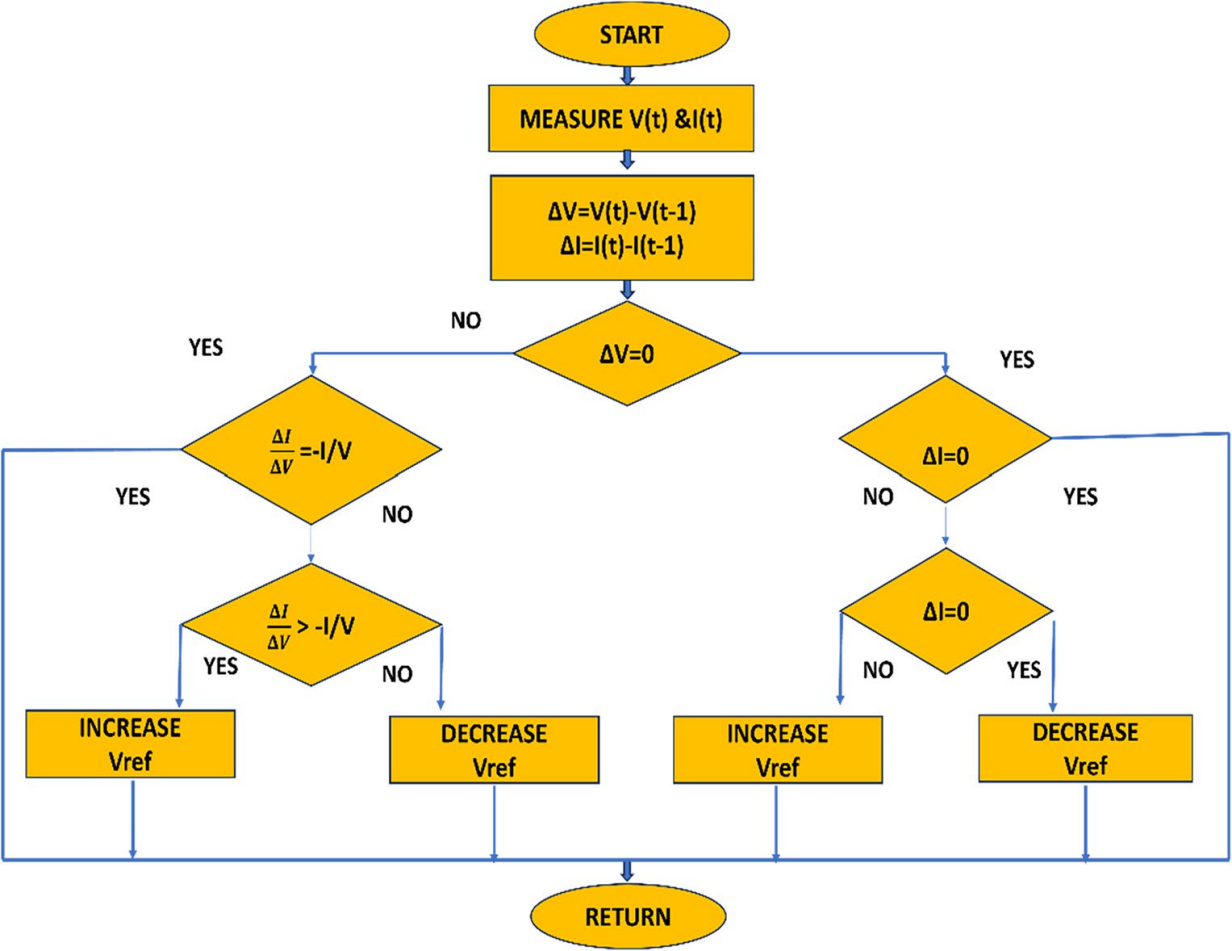
Shows the flow diagram of INC.

INC MPPT is a widely used technique for MPPT in PV systems. It functions by comparing the INC (dI/dV) and instantaneous conductance (I/V) of the PV module^26^. The main idea behind this technique is to compute the power of the PV module in relation to voltage and ensure that the result is balanced to zero. This equilibrium condition is important because it shows that the system is operating at the MPP, where the output power is maximum. The INC MPPT algorithm effectively and dynamically tracks the MPP, maximizing the PV system's energy extraction under fluctuating environmental and load conditions by continuously monitoring and adjusting the operating voltage or current based on the balance between instantaneous and incremental conductance^27^. As a result of its capacity to raise the general effectiveness and performance of solar PV systems, the INC MPPT technology has become quite well-liked. The research flow diagram of the INC controller is given in Fig. 2.

#### Working principle of INC MPPT controller

As a result of employing the differential of the operating point, or dp/dv, the INC algorithm exhibits a remarkable ability to follow the MPP in spite of continuously changing climatic conditions. This method is predicated on the fundamental notion that the power derivative with respect to voltage or current equals zero at the maximum power point^28^. As the weather changes, the INC algorithm dynamically adjusts the operating point in the direction of the MPP. It is crucial to emphasize that, on the left side of the MPP, power exhibits an increasing trend with voltage, while on the right side of the MPP, power exhibits a declining trend with voltage. The INC algorithm takes advantage of these unique tendencies to maximize system power extraction, ensuring improved energy efficiency and performance. Thus, the INC algorithm can be defined mathematically in Eq. (1) as follows:

1 $$ \begin{aligned} & \frac{dP}{{dV}} = 0\quad {\text{At the MPP}} \\ & \frac{dP}{{dV}} > 0\quad {\text{To the left of MPP}} \\ & \frac{dP}{{dV}} < 0\quad {\text{To the right of MPP}} \\ \end{aligned} $$

Because it offers the following advantages, the INC MPPT controller is a popular choice for optimizing energy extraction from PV solar systems.

#### Advantages of INC MPPT controller

The MPP can be efficiently tracked by the INC MPPT controller across a range of weather conditions and load variations. Ensuring that the PV system is running at the MPP raises energy conversion efficiency. The INC algorithm can readily adapt to variations in temperature and solar irradiation because of its quick reaction time. The controller's reactivity allows it to maintain system operation at the MPP even in the face of fast environmental changes. The INC MPPT controller accurately tracks the MPP by comparing the instantaneous conductance (I/V) with the incremental conductance (dI/dV)^29^. The technology continually extracts the greatest amount of power from the PV array thanks to this accurate tracking.

#### Disadvantages of INC MPPT controller

Unfortunately, the INC MPPT controller has a number of limitations and disadvantages that need to be considered: Oscillations around the MPP may occasionally be seen on the INC MPPT controller, particularly when the PV system is running under dynamic or quickly changing conditions^30^. These oscillations have the potential to cause minor power losses and possible damage to power electronic components by forcing an unnecessary operating point transition. Partial shade situations might provide challenges for INC MPPT controllers since they rely on comparing incremental and instantaneous conductance to calculate the MPP. The controller may become confused by many MPPs brought on by partial shadow, which could cause it to pursue less-than-ideal locations or become stuck in local peaks. Because the INC method calculates the derivative of power with respect to voltage, errors in current and voltage measurements may occur. Noisy data can result in incorrect derivative computations, making it more difficult for the controller to properly follow the MPP. Temperature variations may affect the INC algorithm's performance, which could affect how accurately it tracks the MPP^31^. Calibration or temperature correction techniques can be required to counteract this effect.

### Introduction to perturb and observe

**Figure 3:**
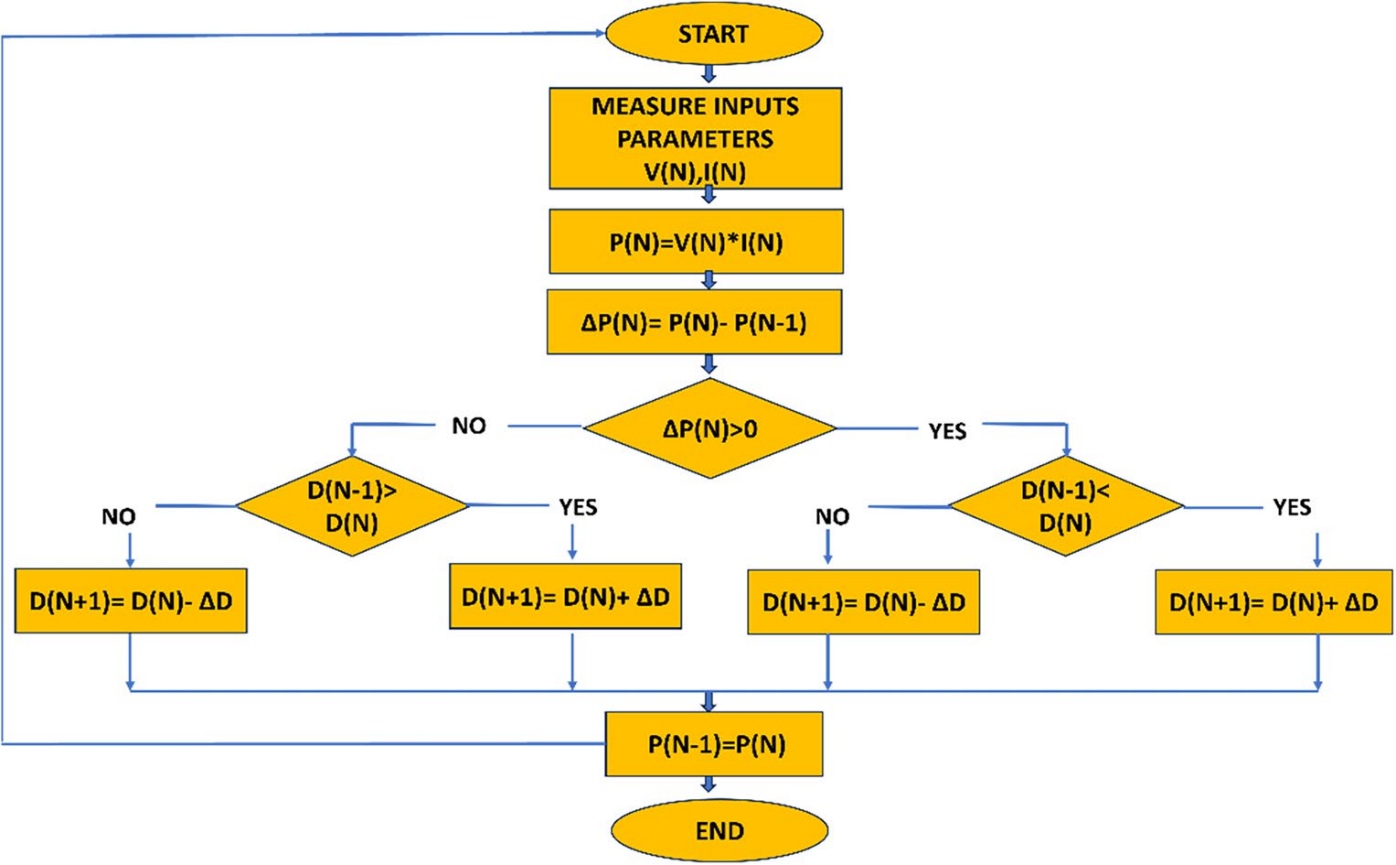
Shows the flow diagram of P&O^33^.

In photovoltaic systems, one of the most used MPPT algorithms is the P&O algorithm. Its basic idea is to gradually alter the PV system's operating point while closely observing how the power output changes in response. The operating point is changed to improve power output after reaching the maximum power point^32^. Due to its simplicity and ease of implementation, the P&O algorithm is preferred. It is important to keep in mind, though, that it has a somewhat slow tracking speed and that it might not reach the global maximum power point. The flow diagram of the P&O controller is given in Fig. 3.

#### Working principle of P&O

The first step of the method is to establish the PV system's initial operating point. Any point on the PV module's current–voltage (I–V) curve can serve as this. The algorithm modifies the voltage or duty cycle (if the system is based on a DC–DC converter) to slightly disrupt the operating point. Usually tiny in size, this perturbation is selected according to the desired pace of convergence and the properties of the system. The algorithm calculates the power output change upon perturbation^26^. At the initial functioning point, it contrasts the new and past power outputs. The program then decides what to do based on the power output change that was noticed. The program keeps perturbing in the same direction to keep going in the direction of the maximum power point if the power grows. In the event that power drops, the algorithm returns to the highest power point by reversing the perturbation direction. Until the algorithm determines the operating point where the power output is at its maximum, the perturbation and observation processes are repeated iteratively. The algorithm determines that the system has reached the maximum power point when it observes a change in power output that is very close to zero or below a predefined threshold^34^. After that, the P&O algorithm modifies the operating point to remain at the maximum power point and keeps an eye on the system all the time to ensure peak power extraction—especially in the event of changing load or weather.

It's mandatory to note that the simplicity of the P&O algorithm allows for easy implementation, but it also brings certain limitations, such as oscillations and sensitivity to partial shading or dynamic weather conditions.

#### Advantages of P&O

Due to its ease of implementation, the P&O method is a popular option for small-scale photovoltaic systems with constrained computing and hardware resources. The technique is economical for realistic MPPT implementations since it requires little extra hardware and only uses simple sensors to detect changes in power output^35^. P&O functions in real-time, continuously modifying the PV system's operating point to track the maximum power point in a variety of environmental circumstances.

#### Disadvantages of P&O

Around the maximum power point, P&O may behave oscillatory. This would force the system to continually alternate between higher and lower power points, which would result in less-than-ideal efficiency. P&O's incremental perturbation strategy may cause a delayed convergence to the maximum power point, particularly when the weather is changing quickly. Due to its reliance on tiny perturbations, the algorithm can be inaccurate or inefficient, particularly when the PV system is subjected to partial shadowing or unsuitable climatic conditions^36^. Sometimes, P&O will converge to a local maximum power point rather than the global maximum, which will reduce the total power output.

### Introduction to incremental conductance—particle swarm optimization (INC-PSO) hybrid MPPT

In order to maximize the efficiency of PV systems, MPPT is a crucial approach that tracks the operating point that yields the maximum power output from the PV module under a variety of environmental conditions. PSO and INC are two popular MPPT algorithm techniques. Every approach has advantages and disadvantages of its own. While the PSO method is competent at locating the GMPP, it can occasionally be slow. The INC approach is quick and effective, but it can become stuck in local maxima. The INC and PSO algorithms are combined in a hybrid MPPT algorithm that has been developed to overcome the shortcomings of separate approaches. The hybrid MPPT algorithm INC-PSO seeks to capitalize on the advantages of both INC and PSO to achieve faster and more accurate tracking of the GMPP under dynamic irradiance conditions.

#### Working principle of INC-PSO hybrid MPPT

The INC-PSO hybrid MPPT algorithm combines the PSO algorithm's global search power with the INC algorithm's local search capabilities. The PSO method is utilized to refine the operating point and converge to the GMPP, whereas the INC algorithm does a quick initial search to find the area around the GMPP^37^.

The INC-PSO hybrid MPPT algorithm operates as follows:

1. *Initialization* Initialize the INC and PSO algorithms with appropriate parameters.
2. *Measurement* Measure the current (I) and voltage (V) of the PV module.
3. *INC Algorithm* Calculate the incremental conductance (dI/dV) using the measured I and V values.
4. *PSO Algorithm* Update the positions and velocities of the particles in the PSO swarm based on the current operating point and the global best position.
5. *Operating Point Adjustment* Adjust the duty cycle of the DC–DC converter based on the output of the INC algorithm and the position of the best particle in the PSO swarm.
6. *Convergence Check* Check if the convergence criteria are met. If not, repeat steps 2–5.

#### Advantages of INC-PSO hybrid MPPT

The INC-PSO hybrid MPPT algorithm offers several advantages over traditional MPPT methods^38^, including:

1. *Faster tracking speed* The INC algorithm provides fast initial searching, while the PSO algorithm ensures convergence to the GMPP.
2. *Improved accuracy* The combination of INC and PSO algorithms enhances the accuracy of GMPP tracking, especially under rapidly changing irradiance conditions.
3. *Reduced sensitivity to noise* The INC algorithm is less sensitive to noise compared to other MPPT methods.
4. *Robustness to partial shading* The INC-PSO hybrid MPPT algorithm is more robust to partial shading conditions compared to traditional methods.

#### Disadvantages of INC-PSO hybrid MPPT

The INC-PSO hybrid MPPT algorithm also has some disadvantages compared to traditional methods^39^:

1. *Increased computational complexity* The PSO algorithm introduces additional computational complexity compared to simpler MPPT methods.
2. *Parameter tuning* The performance of the INC-PSO hybrid MPPT algorithm depends on the tuning of parameters for both INC and PSO algorithms.
3. *Potential instability* Under certain conditions, the INC algorithm may cause oscillations around the MPP, which can affect the stability of the PV system.

### Introduction of perturb and observe-particle swarm optimization (P&O-PSO) hybrid MPPT

MPPT algorithms are crucial for ensuring that PV systems operate at their maximum power output. The other innovative approach in this domain is the integration of the P&O algorithm with PSO, creating a P&O-PSO hybrid MPPT system.

#### Working principle of P&O-PSO

The P&O-PSO hybrid MPPT algorithm amalgamates the simplicity of the P&O algorithm with the global optimization capabilities of PSO. The Perturb and Observe component is responsible for perturbing the operating point of the PV system, observing the resulting power change, and determining the direction in which the power increases. This local optimization method is complemented by the global search capabilities of PSO, which adjusts the step size and perturbation direction dynamically based on the collective intelligence of particles in the swarm. The PSO component enhances the P&O algorithm by providing a more robust and adaptive mechanism for tracking the MPP under various operating conditions, including changes in solar irradiance and temperature.

#### Advantage of P&O-PSO

The integration of PSO with P&O improves the accuracy of the MPPT algorithm, ensuring a more precise and reliable tracking of the MPP under diverse environmental conditions. Additionally, PSO's ability to explore the solution space globally enhances the P&O algorithm's local optimization, making the hybrid approach suitable for complex and dynamic PV system operating conditions. The P&O-PSO hybrid is adept at adapting to changes in solar irradiance and temperature, providing a responsive solution for PV systems in fluctuating environments. Finally, the global search capabilities of PSO contribute to faster convergence towards the MPP, minimizing tracking time and maximizing the energy harvesting efficiency of the PV system^40^.

#### Disadvantages of P&O-PSO

The incorporation of PSO may introduce additional computational overhead, potentially impacting real-time performance, especially in resource-constrained applications. On the other hand, tuning the parameters of both P&O and PSO components is critical, and the performance of the hybrid system may be sensitive to the initial conditions and the selected parameter values. Also, implementing and fine-tuning a P&O-PSO hybrid MPPT algorithm may require a deep understanding of both P&O and PSO techniques, potentially posing challenges for practitioners^41^.

## Artificial intelligence (AI) based MPPT techniques

AI-based controllers represent a cutting-edge paradigm in the optimization of solar energy systems, revolutionizing the field of MPPT. These controllers leverage advanced techniques such as ANN, FLC, PSO, and ANFIS. It introduces a sophisticated layer of intelligence to the MPPT process. By harnessing the power of AI, these controllers autonomously adapt to complex and dynamic environmental conditions, offering a higher degree of accuracy and efficiency in tracking the MPP of PV systems. The integration of AI-based controllers contributes to improved performance, adaptability, and robustness, positioning them as pivotal tools in the quest for enhanced solar energy harvesting. A nuanced comprehension of these AI-based approaches is indispensable for researchers and engineers seeking to propel the advancement of intelligent MPPT strategies in the realm of renewable energy systems.

### Introduction to artificial neural network (ANN)

**Figure 4:**
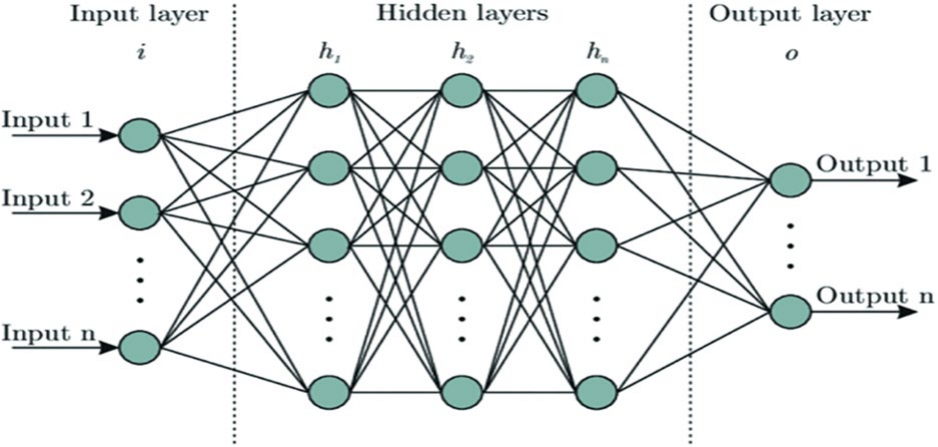
Shows the flow diagram of ANN^42^.

A neural network is a highly efficient, parallel-distributed processor capable of acquiring and utilizing experiential knowledge. Like the human brain, it learns through a process of acquiring synaptic weights, which represent the inter-neuron connection strengths and store the acquired knowledge. Neural networks are well-suited for handling large and complex systems with numerous interconnected parameters, as they prioritize important inputs and filter out less significant data. Among the various learning algorithms for neural networks, the most popular and powerful one is back-propagation and its variants. This error-correction learning rule involves two passes through the network's layers: a forward pass and a backward pass. Neural networks possess remarkable capabilities in processing data, resembling the functioning of the human brain. They excel in handling intricate systems by focusing on crucial inputs and can learn from experiences using advanced learning algorithms such as back-propagation. Artificial neural networks offer numerous advantages, encompassing robust functionality, rapid convergence, resilience to non-linear systems, and the capability for offline training. The flow diagram of the ANN controller is given in Fig. 4.

#### Working principle of ANN

An ANN consists of artificial neurons that act as nodes in a weighted directed graph. The connections between the inputs and outputs of neurons are made by the directed edges with set weights. The ANN receives input signals from external sources in the form of image-representing patterns and vectors. The strength of the inter-neuronal connections throughout the entire ANN is then determined by multiplying these inputs by matching weights^42^. The computation unit handles the weighted inputs, computing their sum. Subsequently, the output is activated by making it non-zero and scaled to the system's desired response using a bias or other mechanisms. The bias input holds a weight of one, remaining consistent across all connections. The total number of weighted inputs may vary from zero to infinity. The activation function plays an important role in transmitting the sum of weighted inputs, in some cases, limiting the response to the desired range. The activation function constitutes a collection of transfer functions, collaboratively achieving the intended effect. Numerous types of activation functions exist, with linear and nonlinear sets being the most prevalent among them.

Supervised learning is a well-established learning paradigm employed in neural networks, wherein the network is guided to acquire knowledge by leveraging known target outputs. Throughout the supervised training phase, the optimization algorithm Stochastic Gradient Descent (SGD) is utilized to iteratively update the values of the hidden units (c) and weights (w), with the main goal being the minimization of the error function E. This error function measures the difference between the target outputs and the predicted outputs produced by the network, which motivates iterative parameter adjustments to boost the network's predictive power and performance. The iterative nature of SGD facilitates the network's convergence towards local minima on the error surface, hence enhancing the model's accuracy and generalization when presented with new input data^43^. The rule for center learning is given in Eq. (2)

2 $$ \begin{aligned} & hxy\left( {t + 1} \right) = hxy\left( t \right) - \eta {1}\partial {\text{E}}/\partial {\text{hxy}} \\ & {\text{For}}\,{\text{ x}}\,{\text{ from}}\,{ 1}\,{\text{ to}}\;{ 3}, \, \;{\text{y}}\;{\text{ from }}\;{1}\;{\text{ to}}\;{\text{ n}}. \\ & wx\left( {t + 1} \right) = wx\left( t \right) - {\upeta }2\partial {\text{E}}/\partial {\text{WX}} \\ \end{aligned} $$

where the cost function, E = 12∑(kd − k)2, k^d^ shows the actual MPP voltage.

#### Learning technique of ANN

##### Advantages of ANN

ANNs offer a powerful and flexible tool for various tasks due to their numerous advantages. ANNs are well suited to handling issues with detailed patterns and interactions because they can model complex, non-linear relationships between inputs and outputs. ANNs can update their internal parameters (weights and biases) over time to increase performance by learning from data. ANNs' capacity to learn from examples enables them to generalize and produce precise forecasts on brand-new, untried data^44^. The parallelization of ANN computations allows them to process several inputs at once. When used in hardware implementations, this parallel processing capacity can result in significant speed increases for some tasks. ANNs can handle noisy or incomplete data, as they can learn to recognize relevant patterns despite uncertainties in the input. ANNs eliminate the need for laborious manual feature engineering by automatically learning to extract pertinent features from raw data. Large and complicated datasets can be handled by ANNs by scaling. Deep Learning architectures have demonstrated remarkable performance in handling vast amounts of data. ANNs can be updated and retrained to adapt to changing data distributions or new requirements, allowing them to remain relevant in dynamic scenarios. ANNs can be integrated with other algorithms or techniques to enhance their capabilities and achieve even more sophisticated outcomes.

##### Disadvantages of ANN

ANNs have many benefits, but they also have significant drawbacks and restrictions. ANNs are frequently regarded as "black boxes," which denotes their lack of interpretability. It can be difficult to comprehend how a network makes a certain choice or prediction, particularly in deep and complicated topologies. Overfitting is a condition in which an ANN performs incredibly well on training data but is unable to generalize to new, unknown data. When a network gets overly complicated or when there is not enough or noisy training data, overfitting may happen. It can be costly and time-consuming to train large-scale ANNs, especially deep neural networks, which require specialized hardware like GPUs or TPUs. ANNs heavily rely on large amounts of labeled training data. Acquiring and preparing such datasets can be laborious and costly, especially in domains with limited data availability. Selecting an appropriate architecture and tuning hyper-parameters can be challenging and often requires trial and error, making the design process time-consuming. Deep Learning models can have significant memory requirements, making them less suitable for deployment in resource-constrained environments.

### Introduction to adaptive neuro-fuzzy inference system (ANFIS)

By fusing the benefits of ANN and FLC, the ANFIS controller creates a controller with outstanding capabilities. These controllers are especially well-suited for nonlinear systems like SPV modules because they exhibit rapid reactivity and excellent efficiency under a variety of weather conditions^45^. The benefit of the ANFIS controller is that it can reduce the complexity associated with conventional fuzzy controllers by automatically designing rules and membership functions during the learning and training process ^46^. These issues are successfully handled by the ANFIS controller's neural network and fuzzy logic integration. The ANFIS controller effectively handles the variability in irradiance and temperature conditions by utilizing the adaptability and learning capacity of neural networks and the interpretability of fuzzy logic. As a result, it ensures that SPV modules respond quickly and work at their best regardless of the weather. A controller with improved accuracy, robustness, and efficiency is produced by this special fusion of neural networks and fuzzy logic, making it an appealing option for managing solar photovoltaic systems.

#### Working principle of ANFIS

**Figure 5:**
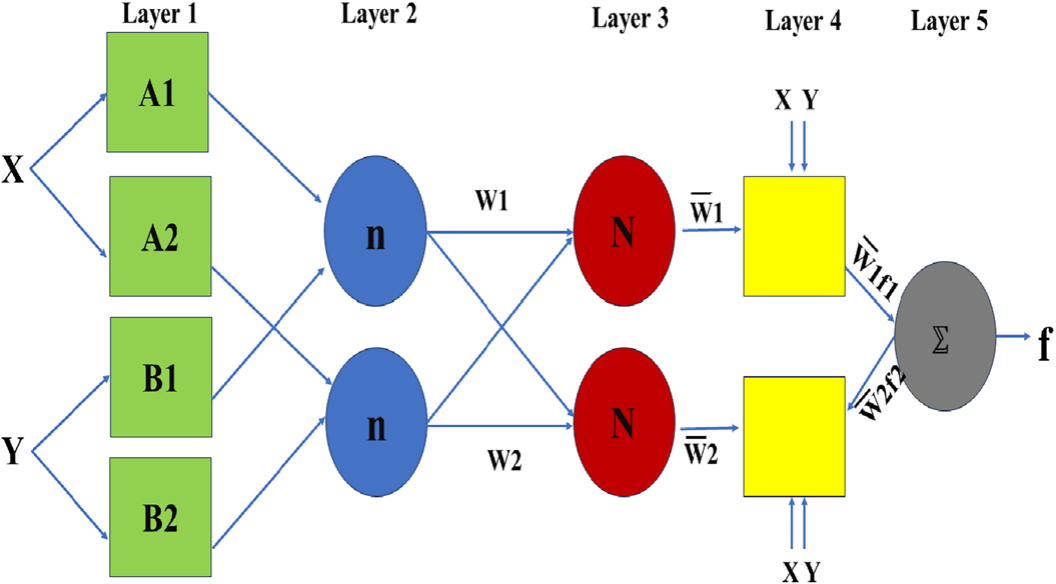
Shows the flow diagram of ANFIS^46^.

Inputs for the ANFIS model include solar irradiation, surrounding temperature, PV array voltage, and PV array current. A flexible and optimized inference system is produced as a result of the vital role the ANN plays in assisting the tuning of the rule table and the membership functions. Through a learning process, the ANFIS inference system successfully optimizes nonlinear functions by efficiently aligning with a collection of fuzzy rule books. It is highly suited for regulating systems with built-in uncertainties and nonlinearity since this combination of ANN and fuzzy logic creates a robust and flexible control system that can handle complicated and interrelated data. The ANFIS controller effectively captures the system's dynamic character, enabling accurate and effective decision-making depending on the inputs^47^. Utilizing the benefits of both ANN and FLC, the ANFIS approach emerges as a comprehensive and sophisticated solution, improving performance and flexibility for a variety of real-world applications. The flow diagram of the ANFIS controller is given in Fig. 5. The fuzzy rule sets for a two-input ()–one output () FIS can be given in Eq. (3): The 1st rule is that if is A_1_ and y is B_1_, then,

3 $$ \begin{aligned} & f_{1} = p_{1} x + q_{1} y + r_{1} \\ & {\text{The}}\;{\text{ 2nd }}\;{\text{rule}}\;{\text{ is }}\;{\text{that }}\;{\text{if}}\;{\text{ x}}\;{\text{ is }}\;{\text{A}}_{2} \;{\text{ and}}\;{\text{ y}}\;{\text{ is}}\;{\text{ B}}_{{2}} \;{\text{ then}}, \\ & f_{2} = p_{2} x + q_{2} y + r_{2} \\ & {\text{And}}\;{\text{ the}}\;{\text{ output}}\;{\text{ function}}\;{\text{ is}}\;{\text{ given}}\;{\text{ by}}\;{\text{ equation}}, \\ & f = \frac{{{\text{w}}_{1} f_{1} + {\text{w}}_{2} f_{2} }}{{{\text{w}}_{1} + {\text{w}}_{2} }} = {\text{w}}_{1} f_{1} + {\text{w}}_{2} f_{2} \\ \end{aligned} $$

#### Advantages of ANFIS

Combining the advantages of fuzzy logic and neural networks, ANFIS can manage complicated and unpredictable systems with ease, which makes it useful in a variety of applications like pattern recognition, decision-making, and control systems. As a result of its ability to adapt and learn from data, ANFIS is able to fine-tune its settings and improve its performance depending on the particular issue it is trying to solve. Through a learning/training process, ANFIS may build fuzzy rules and membership functions automatically, eliminating the need for human rule construction, which can be difficult and time-consuming. ANFIS can offer interpretability in the form of fuzzy rules and membership functions, in contrast to standard black-box machine learning models, enabling users to comprehend the decision-making process, and offering insights into the behavior of the system. ANFIS can handle complicated and non-linear systems because it is effective at modeling and approximating nonlinear interactions between inputs and outputs. When new information becomes available or the system's behavior changes, ANFIS can be updated and retrained, which enables it to continue to be useful in dynamic situations.

#### Disadvantages of ANFIS

It can be difficult to set up an ANFIS model, especially for people who are unfamiliar with both neural networks and fuzzy logic. It can take a while to define suitable fuzzy sets, membership functions, and rules, and domain knowledge is necessary. For ANFIS to effectively optimize its parameters, a large amount of training data is often needed. Large and representative datasets can be difficult to acquire and occasionally impractical. Particularly for big and complicated models, the ANFIS training procedure can be computationally taxing. Speeding up the training process might require the use of strong computing resources. The general behavior of the model can be difficult to understand even with the help of fuzzy rules and membership functions in ANFIS, especially in complicated structures with lots of layers and parameters. The initial values of ANFIS's parameter throughout the training process can have an impact on how well it performs. Finding appropriate initial settings may be essential for getting the best results. Similar to fuzzy logic-based systems, ANFIS may not be able to handle data uncertainty or probabilistic models.

### Introduction to particle swarm optimization (PSO)

To effectively monitor the MPP of a PV system, it is advised to integrate a PSO method, which is also referred to as cooperative particles. This PSO technique aims to solve the nonlinear system optimization problem by utilizing a swarm of Np particles. The cooperative particles of the PSO algorithm work together to find and follow the MPP, guaranteeing the PV system's optimal performance^48^.

#### Working principle of PSO algorithm

This strategy is based on a series of five crucial steps :

1. *Initialization* The Np particle swarm is initially initialized by the PSO algorithm with random coordinates and velocities throughout the search space. Every particle is a potential answer to the optimization issue.
2. *Evaluation* This stage involves computing the objective function, which rates how well each particle's solution performs. The objective function of the PV system controller may be based on power output or efficiency.
3. *Update Personal Best (PBest)* Based on how well it performed, each particle updates its unique best-known position (PBest). So far, the particle has found the best solution represented by this PBest.
4. *Update Global Best (GBest)* The best-performing particle in the swarm, as determined by the objective function, is identified as the global best (GBest). This particle's position serves as the optimal solution found by the entire swarm.
5. *Update Velocities and Positions* The particles adjust their velocities and positions using information from both their PBest and GBest. This velocity update allows the swarm to explore the search space effectively and converge toward the MPP.

**Figure 6:**
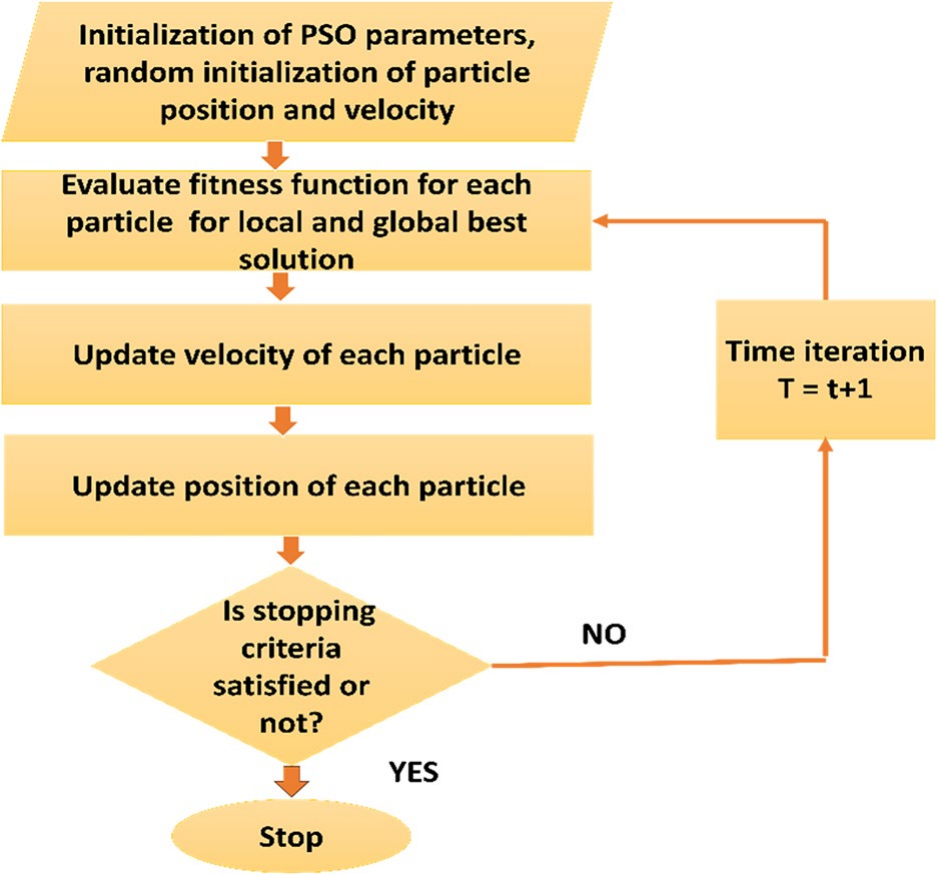
Shows the flow diagram of PSO.

Until a predetermined stopping criterion is satisfied—such as reaching a maximum number of iterations or attaining a desirable level of optimization—these five processes are performed iteratively. By working together, the cooperative particles in the PSO algorithm track the PV system's MPP and guarantee its effective and efficient operation^49^. The flow diagram of the PSO controller is given in Fig. 6.

#### Advantages of PSO technique

The PSO method is a well-liked option for resolving optimization issues since it has a number of benefits. Comparatively speaking to other optimization techniques, PSO is reasonably simple to comprehend and apply. Its premise is social behavior-inspired, making it simple to understand and put into practice. PSO is appropriate for complex, multi-modal optimization problems with numerous optimal solutions dispersed throughout the search space because it may look for global optima. Particularly in the early iterations, PSO can swiftly converge to a nearly optimal solution. It is useful for issues with a vast search space because of this attribute. PSO performs effectively in real-world scenarios with uncertainties because of its robustness in handling noisy objective functions and restrictions. The objective function's gradient or Hessian is not necessary for PSO because it is a derivative-free optimization technique. This qualifies it for non-differentiable optimization.

#### Disadvantages of PSO technique

Although PSO offers many benefits, it also has several drawbacks and restrictions: Premature convergence is a problem in PSO, where the swarm becomes locked in local optima and ignores other interesting areas of the search space. For some difficult situations, this restriction might prevent the algorithm from locating the global optimum. PSO does not ensure convergence to the ideal solution, unlike certain other optimization methods. The features of the problem and the selection of algorithmic parameters can affect the convergence behaviors performance may be impacted by the selection of control parameters, including the swarm size, cognitive and social coefficients, and inertia weight. Finding the right parameter values can be time-consuming and difficult. For discrete or mixed-variable optimization issues, PSO may need to be modified or adjusted from its initial design for continuous optimization problems. PSO's handling of discrete variables can be challenging and can produce unsatisfactory results.

### Introduction to fuzzy logic controller (FLC)

An artificial intelligence system called FLC has emerged that uses fuzzy logic principles to make decisions depending on input and output parameters. Due to its ability to accommodate many input variables and efficiently consider the dynamic nature of the system, FLC outperforms other MPPT approaches in terms of capabilities. Due to this quality, FLC is more adaptable and durable when it comes to maximizing power extraction from solar panels under various environmental circumstances. Fuzzy logic is used in the FLC-based MPPT algorithm to enable adaptive adaptation and response to change solar irradiance, temperature, and partial shade circumstances, which can be difficult for traditional MPPT methods to handle^50^.

#### Working principle of FLC

FLC typically comprises three fundamental stages: fuzzification, rule base, and de-fuzzification. The controller takes variations of error and errors as inputs and produces the duty ratio variation of the DC/DC Boost converter as the output. The inputs of the fuzzy controller are defined by Eq. (1) and Eq. (4):

4 $$ \begin{aligned} & e\left( t \right) = \Delta P\left( t \right) / \Delta V\left( t \right) = \left( {P\left( t \right) - P\left( {t - 1} \right)} \right) / \left( {V\left( t \right) - V\left( {t - 1} \right)} \right) \\ & \Delta e\left( t \right) = e\left( t \right) - e\left( {t - 1} \right) \\ \end{aligned} $$

The error in this case is denoted by "e(t)", which is the ratio of the power change (ΔP(t)) to the voltage change (ΔV(t)) between successive time steps (t and t − 1). With the help of this formulation, the fuzzy logic controller can efficiently ascertain the proper duty ratio modification for the DC/DC Boost converter, guaranteeing optimal power point tracking, and analyze and interpret fluctuations in the system's performance. Our thesis delves into an in-depth examination of the FLC-based MPPT control, exploring its usefulness, benefits, and possibilities for enhancing the effectiveness and flexibility of solar energy systems^51^.

#### Fuzzy rules

The fuzzy rule base is a collection of pre-established rules that aid in figuring out the DC/DC Boost converter's duty ratio depending on error variations and rate of change. The rule base, which is made up of 25 fuzzy control rules, is arranged in Table 1. Each rule is a mix of language variables that determine the output (duty ratio) and the inputs (error and its rate of change).

**Table 1:** Fuzzy rules table.

Output	Change in error, ∆e				
Error, ∆e	NB	NS	ZE	PS	PB
NB	PB	PB	PB	PS	ZU
NS	PB	PB	PS	ZU	NS
ZE	PB	PS	ZU	NS	NB
PS	PS	ZU	NS	NB	NB
PB	ZU	NS	NB	NB	NB

The five different fuzzy levels used for the inputs and output variables are: NB (Negative Big), NS (Negative Small), ZE (Zero), PS (Positive Small) and PB (Positive Big).

Considering the first three rules in the table:

1. IF error is NB (Negative Big) AND error rate of change is NB (Negative Big), THEN the duty ratio is PB (Positive Big).
2. IF the error is NB (Negative Big) AND the error rate of change is NS (Negative Small), THEN the duty ratio is PB (Positive Big).
3. IF error is NB (Negative Big) AND error rate of change is ZE (Zero), THEN the duty ratio is PB (Positive Big).

These rules indicate that when the error is significantly negative (NB) and its rate of change is also significantly negative (NB), the duty ratio of the DC/DC Boost converter should be increased significantly positive (PB). Similarly, in the other two rules, when the error is negative (NB) and its rate of change is either negative small (NS) or zero (ZE), the duty ratio should be adjusted to positive big (PB).

The remaining fuzzy rules in the table follow a similar pattern, representing different combinations of input linguistic variables to determine the appropriate output (duty ratio) to optimize the power extraction from the photovoltaic system. Fuzzy logic allows for precise and flexible control decisions, accommodating various environmental conditions and ensuring effective MPPT operation^49^. The linguistic variables help in representing the system's behavior in human-readable terms, facilitating the interpretation of the control rules, and enhancing the FLC's adaptability and performance^52^.

#### Advantages of fuzzy logic controller

Due to its intrinsic resilience, FLC can withstand fluctuations and disruptions in the working circumstances of the PV system, including shifts in temperature, partial shade, and solar irradiation. By integrating several input variables, FLC allows the controller to consider a greater range of parameters and factors affecting the PV system's operation. PV systems typically exhibit nonlinear behavior due to variations in temperature and irradiation. FLC can successfully manage this non-linearity, enabling precise and effective MPPT tracking in practical settings. The FLC controller employs a rule-based methodology that can include expert knowledge or rules that are data-driven and based on system behavior^53^. This adaptability allows the controller to gather information particular to a certain domain and boost overall performance. Adjust the duty ratio of the DC/DC Boost converter continuously to comply with the fuzzy control rules. By ensuring that the PV system is running at or near its maximum power point, FLC increases energy efficiency and power production. FLC operates in real-time, making it feasible to respond swiftly to changing environmental conditions. For efficient MPPT tracking, this real-time capacity is crucial when weather or load conditions suddenly change.

#### Disadvantages of FLC

Constructing the fuzzy rule base can be challenging and time-consuming, especially for complex systems with multiple inputs and outputs. Expert knowledge or data-driven approaches are required to develop the rules, which might involve trial-and-error iterations for optimal performance. As the number of input variables and fuzzy sets increases, the rule base can become quite large and complex. Managing and maintaining a large rule base may become cumbersome, affecting the controller's computational efficiency.

### Introduction to ANN-PSO hybrid MPPT

ANN-based MPPTs are capable of learning and adapting to complex patterns, but they can be slow and require significant training data. PSO is efficient and can find global optima, but it can be sensitive to parameter settings. A hybrid MPPT algorithm that blends ANN and PSO has been developed to overcome the shortcomings of individual approaches. The goal of the ANN-PSO hybrid MPPT method is to monitor the MPP under dynamic irradiance conditions more quickly and accurately by utilizing the advantages of both ANN and PSO ^54^

#### Working principle of ANN-PSO hybrid MPPT

The hybrid MPPT algorithm known as ANN-PSO combines the ANN's capacity for pattern recognition with the PSO algorithm's capacity for global optimization. The relationship between the power output and operating point of a PV module is taught to the ANN using a huge dataset of PV module I-V curves. Next, the operational point is adjusted using the PSO algorithm, which converges to the MPP^55–57^.

The ANN-PSO hybrid MPPT algorithm operates as follows:

1. *Initialization* Initialize the ANN and PSO algorithms with appropriate parameters.
2. *Measurement* Measure the current (I) and voltage (V) of the PV module.
3. *ANN Processing* Feed the measured I and V values into the ANN to obtain an estimated MPP voltage (V_MPP).
4. *PSO Algorithm* Update the positions and velocities of the particles in the PSO swarm based on the current operating point, the estimated V_MPP, and the global best position.
5. *Operating Point Adjustment* Adjust the duty cycle of the DC-DC converter based on the output of the PSO algorithm.
6. *Convergence Check* Check if the convergence criteria are met. If not, repeat steps 2–5.

#### Advantages of ANN-PSO hybrid MPPT

Comparing the ANN-PSO hybrid MPPT algorithm to conventional MPPT techniques reveals a number of benefits. Based on the patterns it has learned, it offers quick initial searching, and the PSO guarantees convergence to the MPP. Furthermore, the accuracy of MPP tracking is improved by the combination of ANN and PSO algorithms, particularly in situations where irradiance is changing quickly. ANN sensitivity to noise is lower than that of other MPPT techniques. Additionally, compared to conventional techniques, the ANN-PSO hybrid MPPT algorithm is more resilient to partial shading circumstances.

#### Disadvantages of ANN-PSO hybrid MPPT

Comparing the ANN-PSO hybrid MPPT algorithm to other techniques reveals a few more drawbacks. In comparison to more straightforward MPPT techniques, the PSO algorithm adds computing complexity, and the ANN-PSO hybrid MPPT methodology's performance is contingent upon the caliber of the training data that was utilized to educate the ANN. The ANN may not adapt well to real-world situations if it is overfitted to the training set^58^.

### Introduction of FLC-PSO hybrid MPPT

Algorithms for MPPT are essential for maximizing these systems' power output. FLC and PSO are used to create a hybrid MPPT system, which is another creative approach to hybrid MPPTs.

#### Working principle FLC-PSO

The FLC-PSO hybrid MPPT algorithm effectively tracks a PV system's MPP by fusing the adaptive properties of fuzzy logic with the global optimization powers of PSO. A rule-based decision-making mechanism offered by fuzzy logic enables the algorithm to adjust to shifting environmental circumstances. PSO quickly converges towards the MPP by optimizing the fuzzy control parameters^59^. The method continuously adapts the PV system's operating point to dynamic variations in solar irradiance and temperature by iteratively adjusting it based on feedback from the fuzzy controller and PSO's global search capabilities.

#### Advantages of FLC-PSO

Because fuzzy-PSO hybrid MPPT is so good at adjusting to changing environmental factors, it can be used in places where temperatures and sun radiation fluctuate. Furthermore, the PSO component makes it possible for the algorithm to globally explore the solution space, guaranteeing that the MPP is correctly discovered even under challenging and dynamic operating circumstances. As a result, the algorithm continuously runs close to the MPP by constantly modifying the control parameters, which increases energy extraction and boosts system efficiency as a whole^60–62^. Finally, by integrating the benefits of PSO and fuzzy logic, the hybrid approach mitigates the drawbacks of individual algorithms and strengthens the robustness of the MPPT system.

#### Disadvantages of FLC-PSO

The algorithm's hybrid design may result in higher computational complexity, particularly in situations where real-time performance is essential. Furthermore, it can be difficult to fine-tune the settings of FLC and PSO components, necessitating a deep comprehension of the dynamics and performance traits of the system. The algorithm's performance might be affected by the starting parameters and conditions, which could necessitate recalibration in reaction to adjustments made to system elements or external circumstances.

MPPT controllers play a crucial role in optimizing the efficiency of solar photovoltaic systems. Here are the advantages and disadvantages of conventional and artificial MPPT controllers:

1. Advantages of conventional controllers:
  *Simplicity* Traditional MPPT methods are straightforward and easy to implement.
  *Efficiency* They can effectively track a single maximum power point (MPP) under uniform illumination.
2. Disadvantages of Conventional Controllers:
  *Limitations in Partial Shading* Traditional methods struggle to distinguish between local and global peaks in partial shading scenarios, limiting their efficiency.
  *Complexity in Variable Conditions* They may not perform optimally in variable weather conditions.
3. Advantages of AI based controllers:
  *Enhanced Tracking Performance* Advanced MPPT controllers, such as fuzzy logic-based controllers, offer superior tracking performance.
  *Efficiency* They can outperform standard methods in terms of efficiency and performance.
4. Disadvantages of AL based controllers:
  *Increased Complexity* Advanced controllers are more sophisticated, requiring a higher level of technical expertise for installation and maintenance.
  *Cost* They may come at a higher cost due to their complexity and advanced features.
  Advanced MPPT controllers, utilizing soft computing, bio-inspired, or artificial intelligence techniques, offer improved efficiency and performance but require more expertise and investment.

Conventional MPPT controllers are known for their simplicity and ease of implementation, they may struggle in scenarios like partial shading. On the other hand, advanced MPPT controllers provide enhanced tracking performance but come with increased complexity and cost implications. The choice between conventional and artificial MPPT controllers depends on the specific requirements and conditions of the solar photovoltaic system.

Artificial MPPT controllers differ from conventional ones in several key aspects based on the provided sources:

1. Classification:
  *Conventional MPPT Controllers* Conventional MPPT controllers are categorized as traditional methods that are relatively simple and commonly used in solar systems
  *Artificial MPPT Controllers* Artificial MPPT controllers are classified as advanced techniques that leverage AI or hybrid-based methods for enhanced performance and adaptability
2. Technology:
  *Conventional MPPT Controllers* Conventional controllers rely on basic algorithms like P&O and INC for tracking the MPP of solar panels
  *Artificial MPPT Controllers* Artificial controllers utilize advanced technologies such as ANN, ANFIS, or fuzzy logic for more precise and efficient tracking, especially in non-uniform weather conditions and partial shading scenarios
3. Performance:
  *Conventional MPPT Controllers* Traditional methods may have limitations in dynamic weather conditions and partial shading scenarios, affecting their efficiency and adaptability
  *Artificial MPPT Controllers* Artificial controllers offer superior tracking performance, robustness, and adaptability to varying conditions, making them more efficient and effective in optimizing solar system performance
4. Adaptability:
  *Conventional MPPT Controllers* Conventional controllers are generally simpler and may struggle in scenarios like partial shading or rapidly changing weather conditions
  *Artificial MPPT Controllers* Artificial controllers excel in adapting to non-uniform weather conditions, making them more suitable for maximizing power generation in challenging environments
  In essence, artificial MPPT controllers stand out from conventional ones due to their utilization of advanced technologies like AI, which enable them to offer superior performance, adaptability to various conditions, and robustness in optimizing solar system efficiency.

The comprehensive analysis of conventional and artificial intelligence-based controllers provides valuable insights into the nuanced trade-offs between performance and cost across various MPPT algorithms, aiding in informed decision-making for solar power systems. Further analysis of all controllers is given in Table 2.

**Table 2:** Comparative analysis of MPPT controllers.

MPPT method	Cost	Complexity	Response time	Periodic tuning	Stability	Partial shading	Accuracy
Conventional MPPT algorithms							
INC	E	Medium	Varies	No	Yes	No	Medium
P&O	IE	Low	Slow	No	NS	No	Medium
INC-PSO	E	High	Varies	Yes	Stable	Yes	High
P&O-PSO	AF	High	Fast	Yes	Stable	Yes	Medium
FPSO	VE	Low	Fast	No	VS	Yes	High
Intelligent MPPT algorithms							
ANN	E	High	Fast	Yes	VS	Yes	High
ANFIS	E	High	Fast	Yes	VS	Yes	High
ANN-PSO	E	High	Fast	Yes	VS	Medium	Medium
PSO	AF	Medium	Fast	Yes	VS	Yes	Medium
FLC	AF	High	Medium	Yes	VS	Yes	High

## Conclusion

MPPT techniques play a pivotal role in harnessing complete solar energy. As we conclude our exploration into the realm of solar energy systems, it becomes evident that the effective implementation of MPPT strategies is paramount in unlocking the true power and promise of solar energy on a worldwide scale. The presented research aimed to conduct a comprehensive analysis of both individual and hybrid MPPT techniques for efficient solar power generation. The primary focus is on evaluating the efficacy of PV systems in tracking the Maximum Power, aiming to determine the optimal approach for maximizing power production. The study explores various MPPT algorithms, including PSO, FLC, ANN, INC, P&O, and hybrid techniques such as ANFIS. Additionally, combinations of PSO with INC, P&O, Fuzzy, and ANN are examined to provide a comprehensive understanding of their performance in enhancing solar energy system efficiency. The comparison encompasses key parameters such as cost, complexity, response time, stability, partial shading, and accuracy. The findings reveal that among conventional MPPT controllers, FPSO demonstrates superior performance, although at a higher cost. INC-PSO and P&O with PSO follow closely, exhibiting commendable efficiency. INC and P&O present a moderate performance. Furthermore, ANN and ANFIS excel among intelligent MPPT algorithms despite their higher cost. FLC emerges as a strong contender, offering optimal performance at an affordable price despite having a medium response time. Meanwhile, ANN-PSO and PSO deliver moderate performance and affordability.

## Data Availability

The datasets used and/or analyzed during the current study available from the corresponding author on reasonable request.

## Abbreviations

PV: Photovoltaic
MPPT: Maximum power point tracking
AI: Artificial intelligence
DC: Direct current
AC: Alternating current
MPP: Maximum power point
I–V: Current–voltage
ANN: Artificial neural networks
FL: Fuzzy logic
ECs: Environmental conditions
VSR: Voltage source region
CSR: Current source region
ANFIS: Adaptive neuro-fuzzy inference system
INC: Incremental conductance
GMPP: Global maximum power point
PSO: Particle swarm optimization
P–V: Power–voltage
P&O: Perturbation and observation
FPSO: Fuzzy particle swarm optimization
INC-PSO: Incremental conductance-particle swarm optimization
P&O-PSO: Perturb and observe-particle swarm optimization
SGD: Stochastic gradient descent
